# TLTC, a T5 exonuclease–mediated low-temperature DNA cloning method

**DOI:** 10.3389/fbioe.2023.1167534

**Published:** 2023-08-11

**Authors:** Fang Yu, Xia Li, Fei Wang, Yang Liu, Chao Zhai, Wenqiang Li, Lixin Ma, Wanping Chen

**Affiliations:** ^1^ State Key Laboratory of Biocatalysis and Enzyme Engineering, Hubei Province Key Laboratory of industrial Biotechnology, School of Life Sciences, Hubei University, Wuhan, China; ^2^ School of Pharmacy, Qingdao University, Qingdao, China

**Keywords:** T5 exonuclease–mediated low-temperature DNA cloning, T5 exonuclease, molecular cloning, rapid, efficient

## Abstract

Molecular cloning is used in a wide variety of biological and medical research. Here, we developed a rapid and efficient DNA-assembling method for routine laboratory work. We discovered that the cleavage speed of T5 exonuclease is approximately 3 nt/min at 0°C and hence developed a T5 exonuclease–mediated low-temperature sequence- and ligation-independent cloning method (TLTC). Two homologous regions of 15 bp–25 bp compatible with the ends of the vector backbones were introduced into the inserts through PCR. Approximately 120 fmol of inserts and linear vectors was mixed at a molar ratio of approximately 3:1 and treated with 0.5 U of T5 exonuclease at 0°C for 5 min. Then, the mixture was transformed into *Escherichia coli* to generate recombinant plasmids. Single segment and multi-segments can be assembled efficiently using TLTC. For single segment, the overall cloning efficiency is above 95%. Moreover, extra nucleotides in the vectors can be removed during TLTC. In conclusion, an extremely simple and fast DNA cloning/assembling method was established in the present study. This method facilitates routine DNA cloning and synthesis of DNA fragments.

## 1 Introduction

**FIGURE 1:**
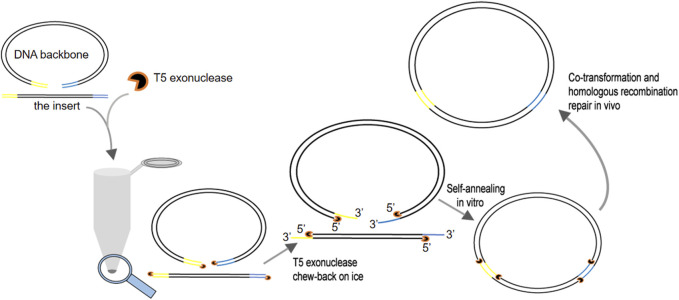
Schematic to illustrate the recommended procedure of the T5 exonuclease–mediated low-temperature DNA cloning method (TLTC).

In comparison with the traditional gene cloning methods utilizing restriction enzymes and ligases, gene assembly strategies based on the DNA repairing and recombination system of *E. coli* are sequence and ligation independent (SLIC) (Li and Elledge, 2007; Zhu et al., 2007; Jeong et al., 2012; Zhang et al., 2014; Motohashi, 2017). The inserts and vector backbones with double- or single-stranded homologous regions flanking the DNA fragments are co-transformed into *E. coil* and joined together seamlessly through the support of the *E. coli* DNA repairing and recombination system. SLIC extremely facilitates the assembly of DNA elements. To improve the cloning efficiency, DNA polymerases with 3′–5′ proofreading activity were chosen to generate single-stranded homologous ends for *in vitro* inter-molecular annealing of DNA fragments. However, the exonuclease activity of these polymerases is relatively low. On the other hand, Gibson assembly (Gibson et al., 2009) uses T5 exonuclease to fulfill this goal. Three enzymes, namely, T5 exonuclease, DNA polymerase, and DNA ligase, are required for this method. T5 exonuclease cleaves linear DNA molecules in the 5′ to 3′ direction (Artymiuk et al., 1997; Garforth and Sayers, 1997; Garforth et al., 2001) and forms long overhangs of hundreds of base pairs for especially annealing between DNA fragments. Fusion DNA polymerase is employed to fill the gaps after annealing of single-stranded DNA compatible regions. Then, Taq DNA ligase joins the DNA fragments. The idea of generating long sticky ends with T5 exonuclease inspired us to develop two site-directed mutagenesis methods, namely, the ICM (*in vitro* CRISPR/Cas9-mediated mutagenic system) (She et al., 2018) and CT5-SDM (FnCas12a and T5 exonuclease-mediated site-directed mutagenesis) (Dong et al., 2020), as well as a cloning method, CT5 (FnCas12a and T5 exonuclease cloning system) (Dong et al., 2021) based on this exonuclease. During this study, we realized that the high activity of T5 exonuclease is a double-edged sword, which causes long single-stranded ends exceeding the length required for inter-molecular annealing of vectors and inserts. For this reason, Gibson assembly deliberately increases the temperature to partially inhibit the activity of T5 exonuclease, hence avoiding overtreatment of the homologous ends flanking the DNA fragments. The optimal temperature of T5 exonuclease is 37°C. Gibson assembly is performed at 50°C because T5 exonuclease remains highly active at 50°C and is deactivated gradually under this elevated temperature. However, the strategy still leads to long single-stranded ends, which in turn leaves long gaps during annealing of compatible ends. These gaps have to be filled by DNA polymerase later. Therefore, an expensive three-enzyme system is required with a long incubation time of 15–50 min. These disadvantages limit its application on routine DNA assembly. Previous reports and the present study indicated that short homologous ends of 15–25 nt are enough to guarantee the efficient recombination of DNA molecules (Zhang et al., 2014). Therefore, we tried to slow down the processing rate of T5 exonuclease further in order to generate short sticky ends. We discovered that T5 exonuclease retained low activity at 0°C and the cleavage speed of T5 exonuclease can be limited to approximately 3 nt/min under this temperature. Hence, we developed a DNA cloning method based on this low-temperature activity of T5 exonuclease instead of inhibiting the enzymatic activity T5 exonuclease through elevating the temperature. Two homologous regions of 15–25 bp compatible with the ends of the vector backbones were introduced into the inserts through PCR. Approximately 120 fmol of inserts and the vector backbones were mixed at a molar ratio of approximately 3:1 and treated with 0.5 U of T5 exonuclease at 0°C for 5 min. Then, the mixture was transformed into *E. coli* to generate recombinant plasmids (Figure 1). This method was named as T5 exonuclease–mediated low-temperature DNA cloning (TLTC) method . This method is fast, efficient, and extremely easy to handle, which is suitable for routine DNA cloning and multi-segments assembling.

## 2 Materials and methods

### 2.1 Strains, plasmids, and media

*E.coli* DH5α, Top10, XL10-Gold, DH10β, and Rosetta blue(DE3) for gene cloning were stored in our lab. The expression vectors pET23a and pET28a were obtained from Novagen (United States). Plasmid pUC57 was obtained from Invitrogen (United States). Plasmids pET28a-gfp, pET28a-mCherry, and pET28a-Kan for multi-segment assembly were stored in our lab. Luria–Bertani (LB) and SOC media for the cultivation of *E. coli* were prepared as described in the Manual of Molecular Cloning. Appropriate antibiotics were added whenever necessary.

### 2.2 Enzymes and reagents

PrimeSTAR® Max DNA polymerase was purchased from TaKaRa (Japan). T5 exonuclease and all restricted enzymes were purchased from New England Biolabs (NEB, United States). The Gibson Assembly® Cloning Kit was purchased from NEB (United States). DNA purification kits were purchased from Omega (United States). All oligonucleotides were synthesized by Shanghai Sangon Biological Engineering Technology & Services Co., Ltd. (Shanghai, China). All other chemicals were analytical reagents.

### 2.3 Preparation of DNA fragments for TLTC cloning

The vector backbones were obtained through either restriction enzyme digestion or PCR. Digestion of the vectors was performed as described in the instructions given by the New England Biolabs (United Kingdom). PCR was performed as described in the manual for PrimeSTAR® max DNA polymerase (TaKaRa, Japan). The PCR products were digested with *Dpn*I to remove the plasmid template, followed by resolving with agarose gel electrophoresis and purifying with the gel purification kit (Omega, United States).

Inserts were gained by PCR with two homologous ends introduced to the PCR products via primer pairs. All the primers used for the amplification of the inserts are listed in Supplementary Table S1.

### 2.4 Recommended method procedure of TLTC

The T5 exonuclease was 10-fold diluted with dH_2_O and stored at 4°C. A 5 µL reaction system was included—3.5 μL of the mixture of the linear vector and insert at a molar ratio of 1:3, 0.5 μL of NEBuffer 4, and 1 μL of diluted T5 exonuclease (0.5 U). The mixture was incubated on ice for 5 min, followed by the addition of 50 μL of *E. coli* DH5α competent cells for transformation.

### 2.5 Transformation of *E. coli* competent cells

*E.coli* DH5α competent cells were prepared according to the previous report (Green and Sambrook, 2018). The transformation efficiency was approximately 3 × 10^8^ cfu/μg. The competent cells (50 μL) were thawed on ice and added to the DNA sample prepared by the TLTC method. The mixture was incubated on ice for 30 min, followed by heat shock at 42°C for 45 s. After cooling the ice for 2 min, 900 μL of the SOC medium was added, followed by shaking at 37°C for 1 h. The samples were diluted and spread on LB agar plates supplemented with the appropriate antibiotics. The plates were incubated at 37°C overnight. The transformation efficiency is defined by the number of colonies, and the recombinant efficiency is evaluated by the percentage of positive colonies against total colonies.

## 3 Result

### 3.1 Optimization of processivity of T5 exonuclease for TLTC

**FIGURE 2:**
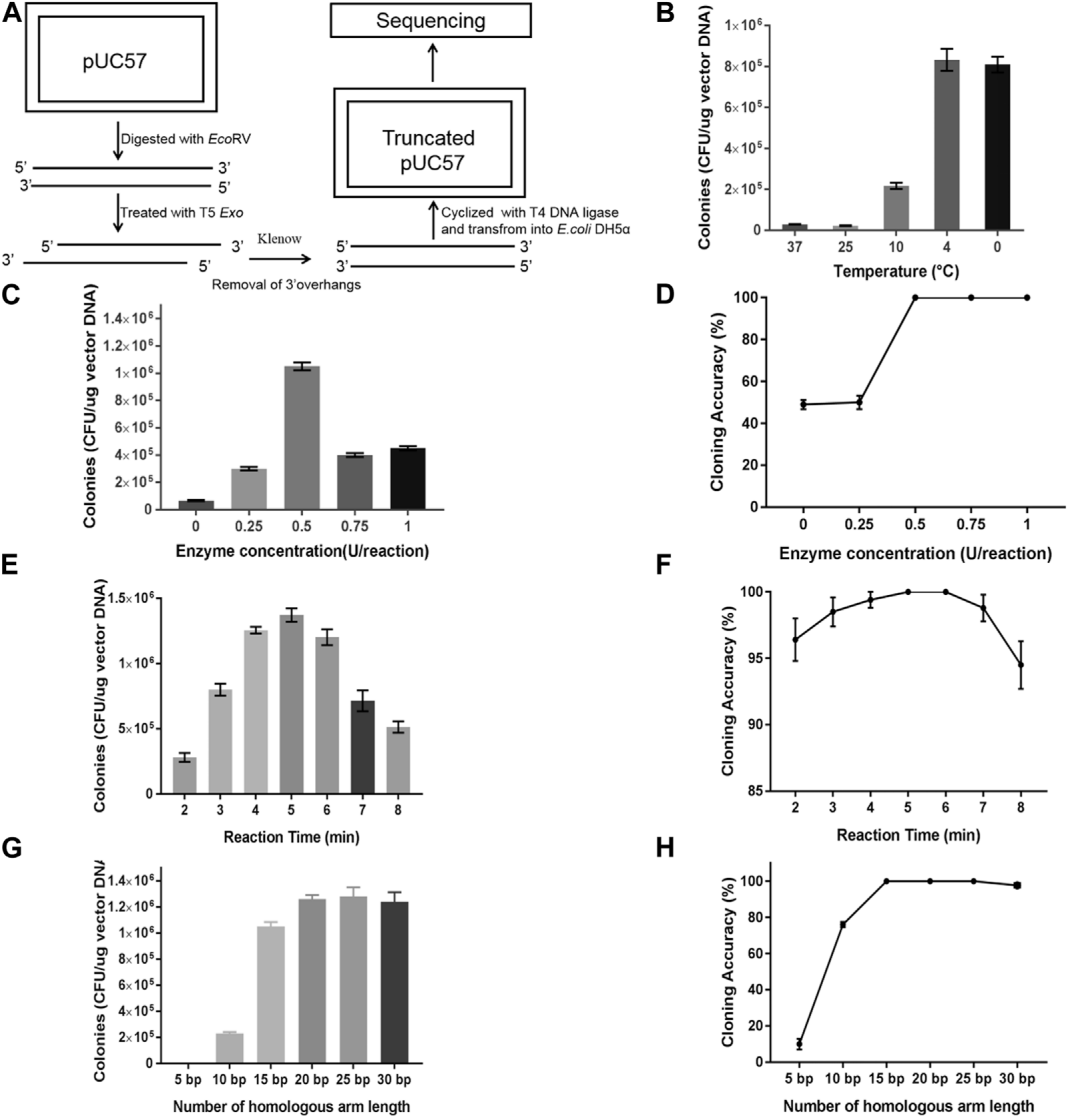
Optimization of processivity of T5 exonuclease for TLTC. **(A)** Evaluating the processivity of T5 exonuclease at 4°C. **(B)** Transformation efficiency of TLTC with the digestion of T5 exonuclease at different temperatures. **(C)** Transformation efficiency of TLTC with T5 exonuclease of different concentrations. **(D)** Recombinant efficiency of TLTC with T5 exonuclease of different concentrations. **(E)** Transformation efficiency of TLTC with T5 exonuclease of different reaction times. **(F)** Recombination efficiency of TLTC with T5 exonuclease of different reaction times. **(G)** Transformation efficiencies of cloning with double-stranded homologous ends of different lengths. **(H)** Recombination efficiencies of cloning with double-stranded homologous ends of different lengths. Results are presented as mean ± SD of three parallel replicates.

According to a previous report, the optimal reaction temperature of T5 exonuclease is 37°C, and it has high activity under this condition. To generate short sticky ends, the sample has be treated for an extremely short time, which is challenging in practical application and lacking of generality. Therefore, we chose to decrease the activity of T5 exonuclease by decreasing the reaction temperature and the amount of enzyme molecules in the reaction system. Therefore, T5 exonuclease was diluted to 1 U/µL, and 1 µL enzyme was added to pUC57 linearized with *Eco*RV (100 ng). The samples were incubated at 0°C for 3–5 min, followed by the addition of Klenow to remove 3′ overhangs formed by T5 exonuclease. Then, the fragments were cyclized with T4 DNA ligase and transformed into *E. coli* (Figure 2A). Colonies were selected randomly for DNA sequencing. The 127-bp sequence around the *Eco*RV site was aligned, and the result indicated that the length of the sticky ends generated by T5 exonuclease increased with the extension of treatment. The average 10-nt ends were formed in 3 min and average 15-nt ends were formed in 5 min (Table 1). This result proves that T5 exonuclease retained a weak activity at 0°C and the short sticky ends could be generated under this condition.

**TABLE 1:** Determining the processivity of T5 exonuclease at 0°C.

3-min Treatment			5-min Treatment		
Remaining length (bp)	Left arm (nt)	Right arm (nt)	Remaining length (bp)	Left arm (nt)	Right arm (nt)
105	15	7	93	15	19
113	7	7	111	12	13
110	13	9	101	13	13
105	15	7	105	15	7
111	9	7	104	14	9
102	10	6	105	14	8
107	13	7	98	20	10
107	14	6	95	14	18
110	5	12	90	13	24
113	7	7	105	14	8
110	7	10	92	23	12
113	7	7	105	14	8

Next, we established the TLTC method based on this condition. To test this method, a gfp fragment was amplified and cloned into pUC57 plasmid. The result indicated that the transformation efficiency of the cloning increased as the temperature decreased and reached a maximum at 4°C (Figure 2B). Interestingly, the result at 0°C was similar to that at 4°C, which indicated that the T5 exonuclease retained activity at 0°C. Since 0°C is easy to gain with ice water mixture, this temperature was used for all the following experiments. The optimal concentration of T5 exonuclease used for the TLTC method was investigated. The result indicated that only 0.5 U of the enzymes was required for a 5 µL reaction system (Figures 2C, D). We also investigated the optimal digestion time of T5 exonuclease. The result indicated that the transformation efficiency reached the maximum after 4–6 min of digestion, and the recombination efficiency was >99% (Figures 2E, F).

The effect of the length of the homologous ends flanking the DNA fragments to the recombination efficiency was tested. First, we mixed the DNA fragments and vector backbones baring the homologous ends of different lengths. The mixture was directly transformed into *E. coli* DH5α. The result indicated that the transformation and recombination efficiencies increased as the compatible ends extended and a minimum 30-nt homologous region was necessary for high recombination efficiency of >95% (Supplementary Figures S1A, B). Meanwhile, we treated the DNA mixture with T5 exonuclease before the transformation. In comparison to the sample without treatment, the number of colonies on the plates increased 10-fold and recombination efficiencies reached >99% with a homologous region of only 15 nt (Figures 2G, H). These results propose that the single-stranded DNA facilitated DNA cloning in TLTC, and a single-stranded homologous region of 15–20 nt was enough for efficient cloning. The results are consistent with previous reports.

The optimal molar ratio of the inserts and vector backbones was investigated. The concentration of the vectors was fixed at 30 fmol as the amount of inserts increased. The result indicated that TLTC worked at a wide range of molar ratios and the best ratio was 1:3 (Supplementary Figure S2), which is consistent with the conventional gene cloning methods.

To evaluate the host strain on TLTC efficiency, *E. coli* DH5α, XL10-gold, Top10, DH10β, and Rosetta blue(DE3) were used to test their compatibility with TLTC. The transformation efficiency of these competent cells was approximately 1 × 10^7^ cfu/μg. As shown in Supplementary Figure S3, DH5α, XL10-gold, and Top10 showed higher efficiency for cloning than DH10β and Rosetta blue(DE3).

### 3.2 Assembly of multiple DNA fragments with TLTC

**FIGURE 3:**
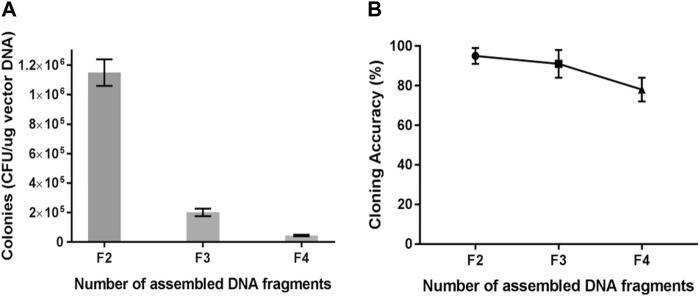
Transformation and recombination efficiency for multi-segment cloning from two to four fragments. **(A)** Transformation efficiency for multi-segment cloning. **(B)** Recombinant efficiency for multi-segment cloning. Results are presented as mean ± SD of three parallel replicates.

Multi-segment assembly was carried out using the TLTC method. All fragments were mixed and treated with T5 exonuclease at 0°C for 5 min, followed by transformation of *E. coli.* For two-fragment assembly, pUC57 provided the vector backbone, while the plasmid pET28a-gfp provided the gfp fragment as the insert. The positive colonies were green under blue light. For the three-fragment assembly, pUC57 provided the vector backbone, while the plasmids pET28a-gfp and pET28a-mCherry provided gfp and mCherry fragments as the inserts. The positive colonies could express gfp and mCherry simultaneously and showed orange fluorescence under blue light. For four-fragment assembly, pET28a-Kan provided the kanamycin-resistant cassette as the third insert. The positive colonies were orange and represented the resistance to kanamycin. The results showed that the transformation efficiency decreased obviously as the number of fragments increased (Figure 3A). On the other hand, the recombinant efficiency still remained high, which was approximately 80% for the four-fragment assembly (Figure 3B).

TLTC is a simplified and versatile system derived from the Gibson assembly. The methods were compared for efficiency with the two-fragment or four-fragment assembly (Supplementary Figure S4). In the two-fragment assembly, when the homologous region was 10 bp, TLTC had greater efficiency than the Gibson assembly, and when the homologous region was extended to 15 bp or 20 bp, TLTC was comparable to the Gibson assembly (Supplementary Figure S4A). In the four-fragment assembly, the Gibson assembly provided greater efficiency (Supplementary Figure S4B).

Promoter, ribosome-binding site (RBS), and terminator strengths determine protein mass fractions and synthesis rates. It is necessary to construct from scratch a full bacterial transcription unit when screening for functional regulatory elements or engineering host cells for improved target protein production. In this research, three DNA fragments (Supplementary Figure S5A): TF1 (containing T7 promoter and RBS), TF2 [containing coding sequence of superfolder GFP (sfGFP)], and TF3 (containing T7 terminator) were assembled into PUC57 with TLTC to construct an entire transcription unit. The TLTC reaction mixture was transformed into Rosetta blue(DE3) competent cells and induced using IPTG. As shown in Supplementary Figure S5B, most of the colonies (>80%) exhibited green fluorescence after 18 h of incubation.

### 3.3 Eliminating extra nucleotides at ends of vector backbone through TLTC methods

**FIGURE 4:**
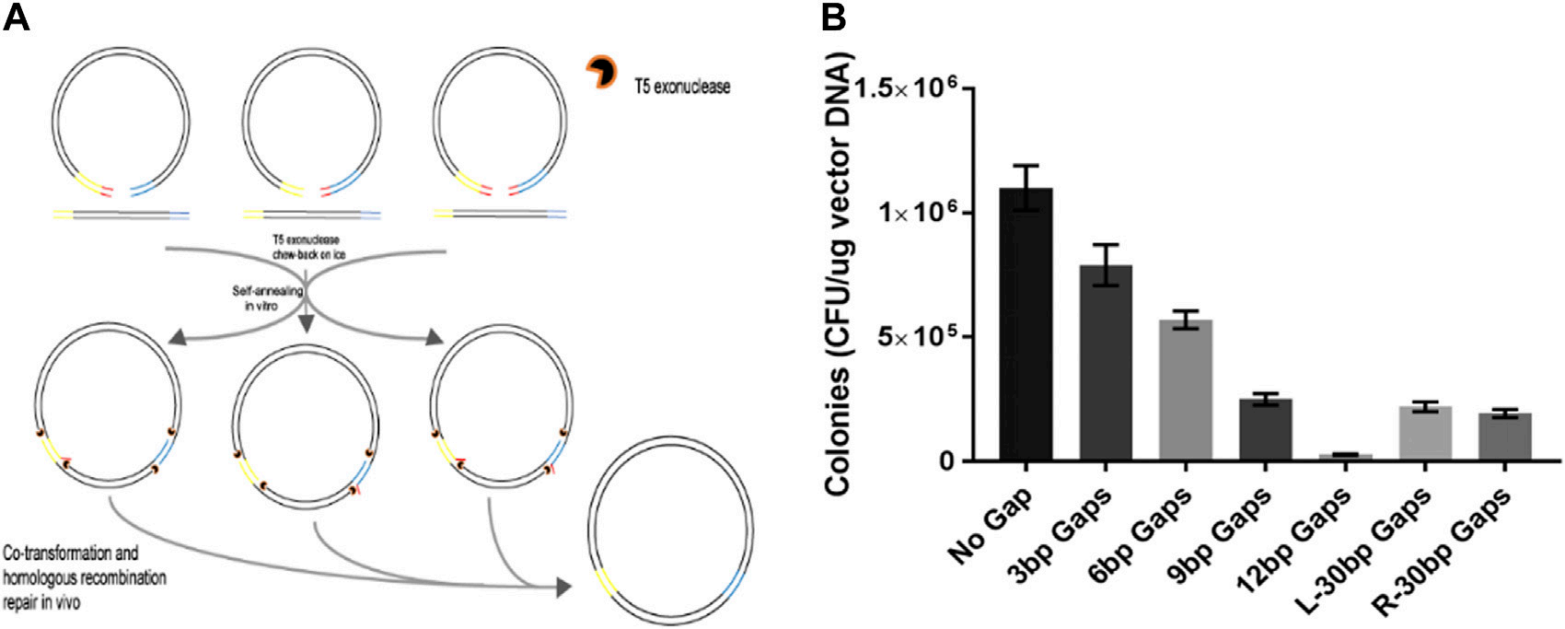
DNA cloning with asymmetric ends between fragments to eliminate extra nucleotides in the vector backbones. **(A)** Schematic to illustrate eliminating extra nucleotides at the ends of the vector backbone using TLTC methods. **(B)** Transformation efficiency of the ligation with asymmetric ends. Results are presented as mean ± SD of three parallel replicates.

A previous report indicated that the short branch was formed if the annealing took place between 3′-ends of the inserts and the distal sequence several nucleotides away from the 3′-ends of the vector backbone (Figure 4A). The branch was later removed by the *E. coli* DNA-repairing system. Hence, short oligonucleotides at the ends of the vector backbone can be eliminated through this asymmetric homologous recombination. In the present study, we tested the efficiency of TLTC in removing the extra nucleotides at the ends of the vector backbones. The result indicated that non-homologous sequences of less than 12 nt were eliminated through TLTC if both ends were not fully compatible with the insert, while non-homologous sequences reaching 30 nt were removed if only one end was not fully compatible (Figure 4B).

## 4 Discussion

With the development of synthetic biology, *de novo* synthesis of large DNA fragments, assembly of multiple DNA elements, and high-throughput cloning of ORFs have become routine laboratory work (Sands and Brent, 2016). Highly efficient DNA assembly methods are necessary to achieve these goals. DNA assembly methods based on the homologous recombination system of *E. coli* are sequence and ligation independent, and seamless. Therefore, it becomes a mainstream cloning strategy nowadays. The key to these methods is forming a circular double-stranded molecule bearing single-stranded gaps of diverse lengths between linear vectors and inserts *in vitro*. After transformation, a RecA-independent mechanism of *E. coli* fills the gaps and ligates the DNA fragments. Previous reports have indicated that the cloning efficiency can be drastically increased by using inserts and vector backbones bearing single-stranded ends to allow *in vitro* inter-molecular annealing (Li and Elledge, 2007). Therefore, how to gain single stranded DNA ends with accurate and homogeneous length is critical to the efficiency of this method. The single-stranded ends can be generated by polymerases or exonucleases. T5 exonuclease has high activity and generates long single-stranded ends (Artymiuk et al., 1997; Garforth and Sayers, 1997; Garforth et al., 2001) to facilitate *in vitro* annealing. However, gaps are left during inter-molecular annealing because the length of these single-stranded ends is highly heterogeneous. Although such gaps are well tolerated in *E. coli*, the maximum gap length that is efficiently repaired by the bacteria is not known. Previous reports have suggested that single-stranded gaps in circular plasmids have a strong negative effect on transformation efficiency (Gibson et al., 2009; Benoit et al., 2016). Therefore, the processivity of T5 exonuclease has to be controlled strictly to minimize the gaps. The Gibson assembly (Gibson et al., 2009) and Nimble cloning (Yan et al., 2020) used 50°C and polymerase. On the other hand, TEDA (T5 exonuclease DNA assembly) chose a condition of 0.04–0.08 U of T5 exonuclease at 30°C in the presence of PEG 8000 (Xia et al., 2019). It has been proposed that PEG 8000 and proper dilution are the keys for generating short homologous regions. Although these methods can guarantee high cloning efficiency, extra reagents were introduced, which increases the cost of cloning. Moreover, the whole procedure becomes time consuming and requires approximately 1 h. We discovered that T5 exonuclease has low-temperature activity and established a novel DNA cloning method—TLTC. The whole procedure can be completed in 5 min and only 0.5 U of T5 exonuclease is required. Moreover, no extra equipment is required for TLTC because the whole procedure can be accomplished simply in ice water mixture. Although T5 exonuclease has similar activity at 4°C and 0°C, we did not recommend 4°C, as it is easier to maintain the reaction mixture at 0°C using the ice water mixture. It is worth noting that the primers for the TLTC method can be limited to 15–25 nt, since there is no requirement for long primers to introduce long homologous regions. Therefore, the cost of primers is low and mismatch of primers to template and primer dimer caused by long primers in PCR can be avoided efficiently. In addition, we have previously combined T5 exonuclease with programmable endonucleases to established highly efficient mutagenesis and DNA cloning methods (She et al., 2018; Dong et al., 2020; Dong et al., 2021). As more and more programmable endonucleases become commercially available, these endonucleases may replace restriction enzymes to generate linear vector backbones in the future (Li et al., 2016; Enghiad and Zhao, 2017). Therefore, establishing a DNA cloning method combining TLTC and programmable endonucleases is an important aspect worthy of intensive study.

In conclusion, we developed an extremely simple and a fast DNA cloning method. This method facilitates routine DNA cloning and assembly of multiple DNA fragments.

## Data Availability

The original contributions presented in the study are included in the article/Supplementary Material; further inquiries can be directed to the corresponding authors.
