# Supplementary material for: TLTC, a T5 exonuclease–mediated low-temperature DNA cloning method
Source: Front Bioeng Biotechnol. 2023 Aug 11;11:1167534. doi: 10.3389/fbioe.2023.1167534 (PMC10457141; doi:10.3389/fbioe.2023.1167534)
Supplement: Supplementary file 1 [file DataSheet1.docx]

**Supplementary**

Supplementary Table 1 primers used in the present study

| **Name of the primer** | **Sequence（5'-3'）** |
| --- | --- |
| **Primers used for testing the length of homologous region for TLTC** | |
| gfp-P57-5-F | TAGATGCCGTTCGCTGTGTCGCA |
| gfp-P57-5-R | CCGATGTGGTGGTGCTCGAGAATTGTGA |
| gfp-P57-10-F | GCATCTAGATGCCGTTCGCTGTGTCGCA |
| gfp-P57-10-R | GGGATCCGATGTGGTGGTGCTCGAGAATTGTGA |
| gfp-P57-15-F | CGAATGCATCTAGATGCCGTTCGCTGTGTCGCA |
| gfp-P57-15-R | GGCCGGGATCCGATGTGGTGGTGCTCGAGAATTGTGA |
| gfp-P57-20-F | CCTCGCGAATGCATCTAGATGCCGTTCGCTGTGTCGCA |
| gfp-P57-20-R | CGACGGGCCCGGGATCCGATGTGGTGGTGCTCGAGAATTGTGA |
| gfp-P57-25-F | CGGTACCTCGCGAATGCATCTAGATGCCGTTCGCTGTGTCGCA |
| gfp-P57-25-R | GCAGTCGACGGGCCCGGGATCCGATGTGGTGGTGCTCGAGAATTGTGA |
| gfp-P57-30-F | GAGCTCGGTACCTCGCGAATGCATCTAGATGCCGTTCGCTGTGTCGCA |
| gfp-P57-30-R | CCTCTGCAGTCGACGGGCCCGGGATCCGATGTGGTGGTGCTCGAGAATTGTGA |
| **Primers for investigating the length of the extra nucleotides eliminated by TLTC** | |
| gfp-P57-gap-3bp-F | gctcggtacctcgcgaatgcatctaGCCGTTCGCTGTGTCGCA |
| gfp-P57-gap-3bp-R | TCTGCAGTCGACGGGCCCGGGATCCGTGGTGGTGCTCGAGAATTGTGA |
| gfp-P57-gap-6bp-F | CGAGCTCGGTACCTCGCGAATGCATGCCGTTCGCTGTGTCGCA |
| gfp-P57-gap-6bp-R | GCCTCTGCAGTCGACGGGCCCGGGAGTGGTGCTCGAGAATTGTGA |
| gfp-P57-gap-9bp-F | ATTCGAGCTCGGTACCTCGCGAATGGCCGTTCGCTGTGTCGCA |
| gfp-P57-gap-9bp-R | CAGGCCTCTGCAGTCGACGGGCCCGGTGGTGCTCGAGAATTGTGA |
| gfp-P57-gap-12bp-F | TGAATTCGAGCTCGGTACCTCGCGAGCCGTTCGCTGTGTCGCA |
| gfp-P57-gap-12bp-R | ATGCAGGCCTCTGCAGTCGACGGGCGTGGTGCTCGAGAATTGTGA |
| gfp-P57-gap-L30bp-F | TTGTAAAACGACGGCCAGTGAATTCGCCGTTCGCTGTGTCGCA |
| gfp-P57-gap-R30bp-R | TGATTACGCCAAGCTTGCATGCAGGGTGGTGCTCGAGAATTGTGA |
| **Primers for multi-segment cloning** | |
| P57-F3-1-R | gagcgctctagaggatacttcac |
| P57-F3-2-F | aagtgaagtatcctctagagcgctcgttgacaattaatcatcggcatagt |
| P57-F3-2-R | GCAGTCGACGGGCCCGGGATCCGATtgatctgtaaccaagcactgat |
| P57-F4-2-R | tgatctgtaaccaagcactgatagt |
| P57-F4-3-F | actatcagtgcttggttacagatcatgttgcagctatcggttctg |
| P57-F4-3-R | GCAGTCGACGGGCCCGGGATCCGATctgatcagcggtgacatcaa |
| P57-T7p-F | cctcgcgaatgcatctagatCCCGCGAAATTAATACGACT |
| T7p-R | tcctcgcccttgctcaccatATGTATATCTCCTTCTTAAAGTTAAAC |
| sfGFP-F | atggtgagcaagggcgag |
| sfGFP-R | tttatacagttcatccatgccca |
| T7t-F | tctgggcatggatgaactgtataaaTAACAAAGCCCGAAAGGAAG |
| T7t-R | cgacgggcccgggatccgatCAAAAcgacgggcccgggatccgatAACCCCTCAAGACCC |

Note: the homologous sequences are indicated in red.


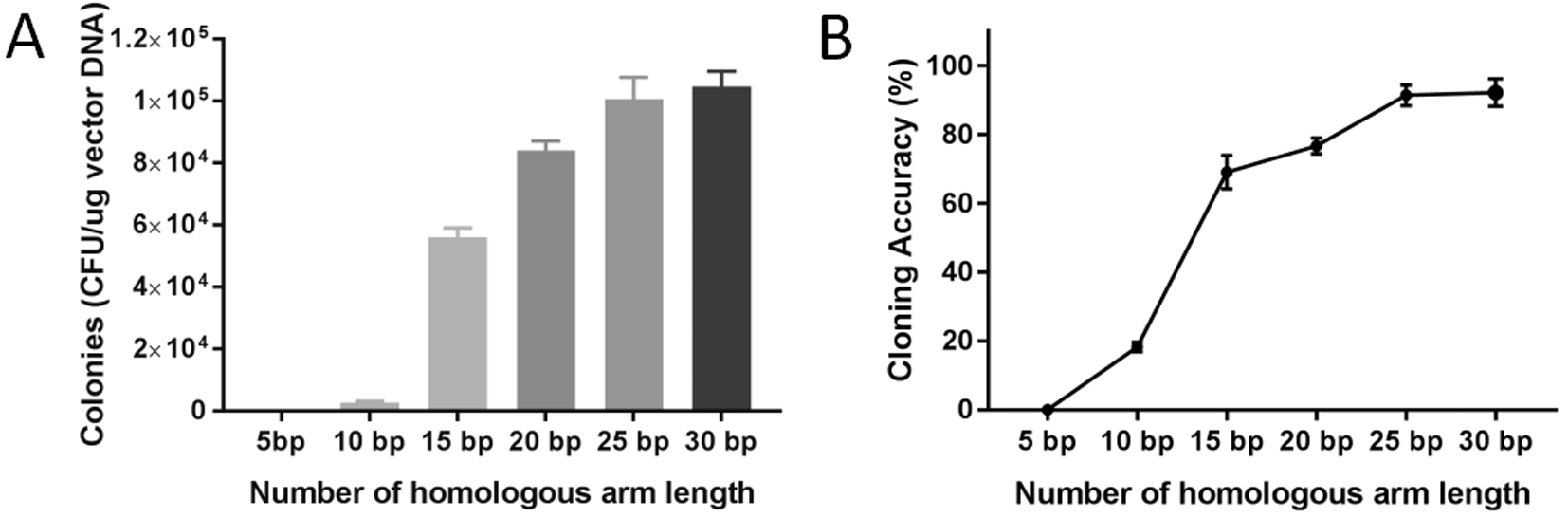


**Figure S1.** The effect of the length of the homologous ends flanking the DNA fragments to the recombination efficiency. (A). the transformation efficiencies of cloning with double-stranded homologous ends of different length; (B). the recombination efficiencies of cloning with double-stranded homologous ends of different length.Results are means ± SD of three parallel replicates.

**
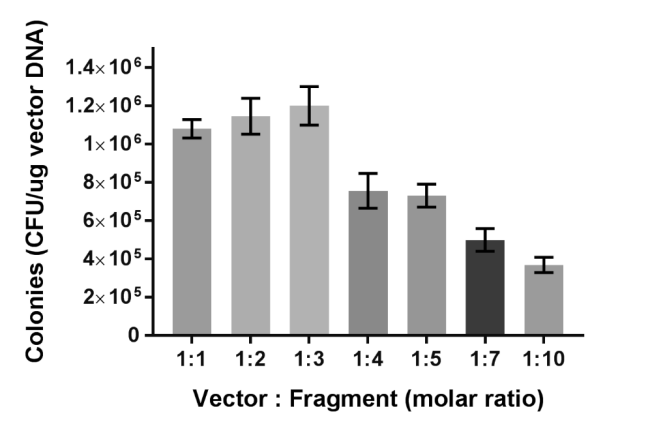
**

**Figure S2.**The molar ratio of the vector and the insert for TLTC. Results are means ± SD of three parallel replicates.

**Figure S3.**The effect of *E.coli* strain on the TLTC efficiency.The TLTC reaction mixture was transformed into competent cells of different *E. coli* strains (The transformation efficiency of these competent cells was about 1×10^7^cfu/μg). Results are means ± SD of three parallel replicates.


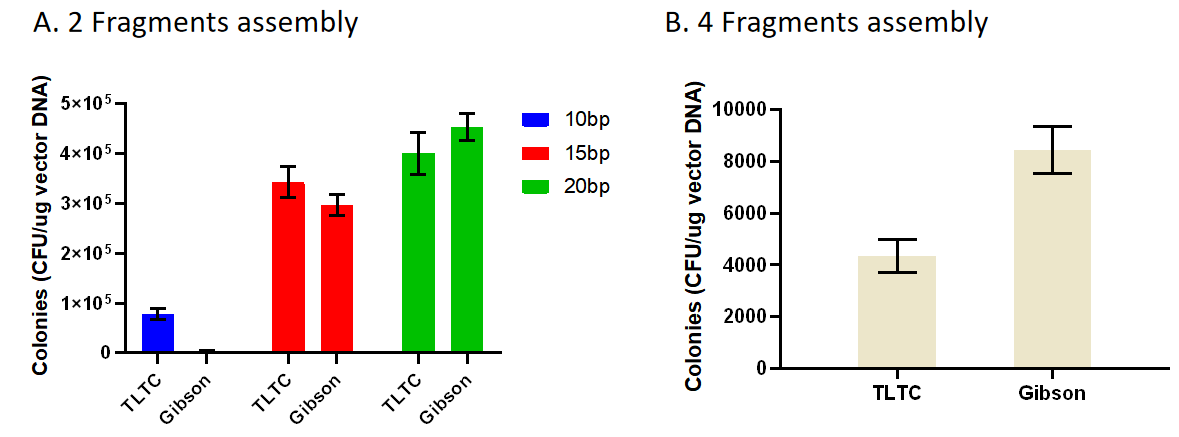


**Figure S4.** Comparation of TLTC and Gibson assembly.(A).TLTC was compared with Gibson for the assembly of two fragments with length of the homologous ends; (B).TLTC was compared with Gibson for 4-fragments assembly. Results are means ± SD of three parallel replicates.


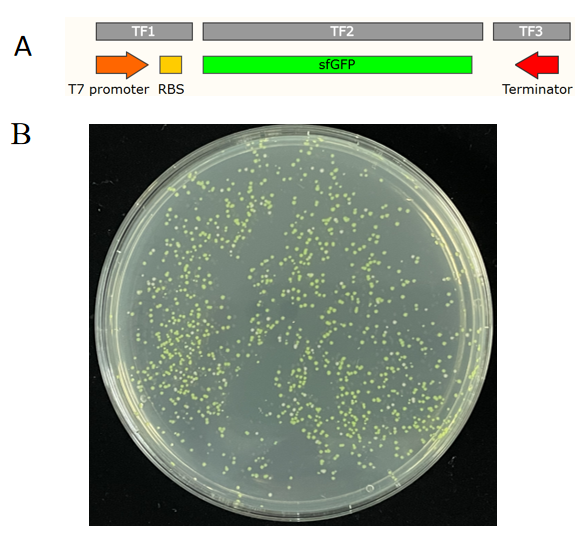


**Figure S5.** Construction an entire transcription unit with TLTC. (A).The elements used in the assembly:TF1 include T7 promoter and RBS, TF2 include coding sequence of superfloder GFP(sfGFP)) and TF3 include T7 terminator; (B).The TLTC reaction mixture was transformed into Rosetta Blue(DE3) competent cells and induced with IPTG, the plate was  incubated at 37 °C for 18 h .
